# Optimal timing of capsular tension ring implantation in pseudoexfoliation syndrome

**DOI:** 10.5935/0004-2749.20210024

**Published:** 2025-02-02

**Authors:** Emrah Ozturk, Abuzer Gunduz

**Affiliations:** 1 Department of Ophthalmology, Malatya Training and Research Hospital, Malatya, Turkey; 2 Department of Ophthalmology, Inonu University School of Medicine, Malatya, Turkey

**Keywords:** Cataract, Capsular tension ring, Phacoemulsification, Catarata, Facoemulsificação, Anel de tensão cap sular

## Abstract

**Purpose:**

The aim of this study was to evaluate the appropriate timing of capsular tension ring implantation in cases of zonular weakness due to pseudoexfoliation syndrome.

**Methods:**

This prospective, comparative study was performed at the Ophthalmology Department of Inonu University, Malatya, Turkey. There were 43 patients included in the study. Group 1 (16 patients) had early capsular tension ring implantation, and group 2 (27 patients) had late capsular tension ring implantation. Patients with pseudoexfoliation syndrome who underwent phacoemulsification surgery, posterior chamber intraocular lens implantation, and capsular tension ring implantation were included in the study. Intraoperative complications and difficulties with either capsular tension ring implantation or cortex removal were evaluated in each eye.

**Results:**

There was no significant difference between the groups in the difficulty of capsular tension ring implantation (p=0.124). The difficulty of cortex removal differed significantly between the groups (p=0.003). Intraoperative complications were observed in 3 patients in group 1 and 11 patients in group 2; the difference between the groups was not significant (p=0.18). Posterior capsule fluctuations were observed in 8 patients (29.5%) in group 2, which resulted in posterior capsule rupture in 2 patients.

**Conclusıons:**

Cortex removal is more difficult with early capsular tension ring implantation, and posterior capsule fluctuations may cause problems with late capsular tension ring implantation. The surgeon must consider the risk‑to‑benefit ratio of early versus late insertion for the optimal timing of capsular tension ring implantation.

## Abstract (pt)

**Objetivo:**

Avaliar o momento apropriado para implante de anel de tensão capsular em casos de fraqueza zonular devida à síndrome pseudoesfoliativa.

**Métodos:**

Este foi um estudo prospectivo e comparativo realizado no Departamento de Oftalmologia da Universidade İnönü. Foram incluídos 43 pacientes, sendo 16 no grupo 1 e 27 no grupo 2. O grupo 1 era composto de pacientes que se submeteram ao implante precoce do anel de tensão capsular, enquanto no grupo 2 os pacientes ti veram implante tardio. Foram incluídos pacientes com síndrome pseudoesfoliativa submetidos à cirurgia de facoemulsificação e ao implante de lente intraocular na câmara posterior e anel de tensão capsular. Em cada olho, foram avaliadas as complicações intraoperatórias e as dificuldades tanto com a implantação do anel de tensão capsular quanto com a remoção do córtex.

**Resultados:**

Não houve diferença significativa entre os grupos quanto à dificuldade de implante do anel de tensão capsular (p=0,124). Ao se comparar as remoções do córtex, observou‑se diferença significativa entre os grupos (p=0,003). Complicações intraoperatórias foram observadas em 3 pacientes do grupo 1 e 11 pacientes do grupo 2; porém, não houve diferença significativa entre os grupos (p=0,18). No grupo 2, observaram‑se flutuações da cápsula posterior em 8 pacientes (29,5%), com ruptura da cápsula posterior em dois deles.

**Conclusões:**

A remoção do córtex é mais difícil no implante precoce do anel de tensão capsular e flutuações da cápsula posterior podem causar problemas no implante tardio do anel de tensão capsular. O cirurgião deve ponderar a relação risco/benefício do implante precoce e tardio ao avaliar o momento ideal para implante de anel de tensão capsular.

## INTRODUCTION

The capsular tension ring (CTR) is designed to stretch the lens capsule and thereby maintain the circular contour of the capsular bag after cataract extraction^(1)^. Since the introduction of the CTR, some of its features have been improved to allow implantation of an intraocular lens (IOL) in the capsular bag based on the surgeon’s evaluation of the compromised zonular fibers^(2)^. CTR usage is becoming more common in cataract surgery. When the CTR is placed in the capsular bag, it supports the area of zonular weakness and redistributes the forces equally over all zonules^(3,4)^. It also stabilizes the capsular bag and the IOL during and after cataract surgery. CTRs are typically used in eyes with presumed or actual zonular weakness or dialysis. These are mostly caused by pseudoexfoliation (PXF) syndrome, high myopia, mature cataracts, ocular trauma, or Marfan syndrome^(2,4^–^6)^.

PXF syndrome is a disease with an unknown etiology that may cause multisystemic and ocular complications^(7)^. PXF is characterized by the production and accumulation of age‑related abnormal fibrillary material in various eye tissues^(8)^. The diagnosis of PXF syndrome is made by observing gray‑white fibrogranular PXF material on the lens anterior capsule or pupil edge during anterior segment examination^(9)^. PXF syndrome is associated with poor pupillary dilation, zonular weakness leading to intraoperative or postoperative lens dislocation, and vitreous loss^(10)^.

Many surgeons feel that the use of a CTR increases success in the case of zonular laxity. The CTR may be implanted at any time following capsulorhexis and hydrodissection or viscodissection. However, much discussion has arisen as to the optimal timing of CTR implantation during cataract removal. This study aimed to investigate the optimal timing of CTR implantation during cataract surgery in patients with PXF syndrome.

## METHODS

This was a prospective, comparative, single‑center study conducted in the Department of Ophthalmology of the Inonu University Faculty of Medicine, Malatya, Turkey, a tertiary care center.

Patients who underwent phacoemulsification surgery, posterior chamber IOL, and CTR implantation were included in the study. All participants gave informed consent before the surgery and the study. The tenets of the Declaration of Helsinki were followed. The study was approved by the Malatya Clinical Research Ethics Committee.

Patients with zonular weakness due to PXF syndrome were included in the study. A complete ophthalmic evaluation was performed in all patients, including assessment of best corrected visual acuity (BCVA), anterior segment evaluation, intraocular pressure (IOP) measurement, and posterior segment evaluation. A B‑scan was performed when the fundus was not visible. BCVA was evaluated using Snellen charts. Patients who had apparent zonulysis and lens dislocation, previous ocular surgery or laser therapy, or a history of any ocular disorder, trauma, or glaucoma were excluded from the study.

The following information was noted in all patients: gender, age, operated eye, preoperative BCVA and IOP, axial length, decision on CTR usage, degree of zonular weakness, iris hook usage, timing and difficulty of CTR implantation, difficulty of cortex removal, and intraoperative complications. The degree of zonular weakness, difficulty of CTR implantation, and difficulty of cortex removal were evaluated subjectively by the same surgeon. The difficulty of CTR implantation and the difficulty of cortex removal were categorized as easy, medium, or difficult. The iris hook was used in patients with poor pupillary dilatation. The iris hooks used for pupil dilatation were also used as capsule retractors in patients with significant zonular weakness.

The decision to insert the CTR was made either preoperatively, when there was apparent phacodonesis, or intraoperatively, when loose zonules were apparent during capsulorhexis. Early CTR implantation was defined as placement of the CTR after hydrodissection or viscodissection and before nucleus removal. Late CTR implantation was defined as placement of the CTR after removal of the nucleus and cortex. The patients were categorized into two groups. Group 1 (16 patients) had early capsular tension ring implantation, and group 2 (27 patients) had late capsular tension ring implantation. In group 2 patients, the capsular bag was filled with a cohesive ophthalmic viscosurgical device before implantation of the CTR.

The same surgeon performed all surgeries with the patient under local or general anesthesia. A 5.5‑mm capsulorhexis and multiquadrant hydrodissection were performed in all eyes. Viscodissection was performed only in group 1 patients using a cohesive ophthalmic viscosurgical device. In group 1, the CTR was placed after hydrodissection and viscodissection. Nuclear phacoemulsification was performed by the quick chop technique. In group 2, the CTR was placed after the nucleus and cortex were removed. A polymethyl methacrylate CTR type 4 (13‑11 mm; Madhu Instruments, New Delhi, India) was inserted into the capsular bag of all eyes with forceps. A foldable posterior chamber IOL was implanted into the capsular bag in eyes with an intact capsular bag or in the ciliary sulcus in eyes with a capsule rupture.

### Statistical analysis

SSPS for Windows statistical software (ver. 22.0; IBM Corp., Armonk, NY, USA) was used for the analyses. The results are expressed as mean ± standard deviation (SD) or median (min‑max). Shapiro‑Wilk tests were used to determine the normality of the distribution of continuous variables. To investigate the differences between the two groups, t‑tests and Mann‑Whitney U tests were used for quantitative data and chi‑square tests were used for qualitative data. A value of p<0.05 was considered to indicate statistical significance.

## RESULTS

A total of 43 patients, 16 in group 1 and 27 in group 2, were included in the study. No statistically significant differences were observed between the groups in gender, age, operated eye, preoperative BCVA and IOP, timing of the decision to use the CTR, degree of zonular weakness, axial length, or requirement for an iris hook (Table 1).

**Table 1:** Demographic and general characteristics of the patients

Characteristic	Group 1	Group 2	P value
Gender Female	7	13	1.00
Male	9	14	
Age (years; mean ± SD)	73.19 ± 12.96	74.19 ± 7.5	0.77
Operated eye Right	9	14	1.00
Left	7	13	
Preoperative BCVA (decimal), median (min‑max)	0.02 (0.01-0.2)	0.01 (0.01‑0.4)	0.92
Preoperative IOP (mmHg; mean ± SD)	13.69 ± 3.70	14.30 ± 3.48	0.49
Axial length (mm; mean ± SD)	23.06 ± 0.81	23.63 ± 1.64	0.09
Decision for CTR Preoperative	4	7	1.00
Intraoperative	12	20	
Zonular weakness Low	7	20	0.09
Medium	6	6	
High	3	1	
Iris hook No	7	13	1.00
Yes	9	14	

SD= standard deviation; BCVA= best corrected visual acuity; IOP= intraocular pressure; CTR= capsular tension ring.

**Figure 1:**
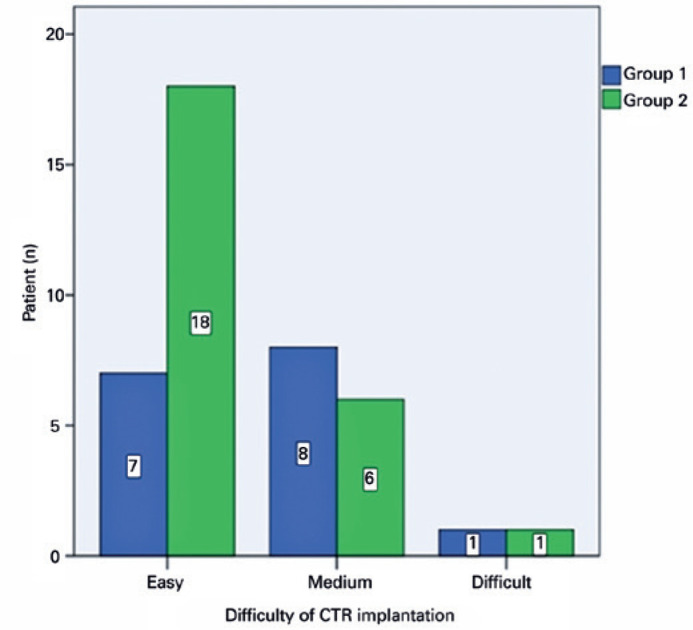
Difficulty of capsular tension ring implantation.

**Figure 2:**
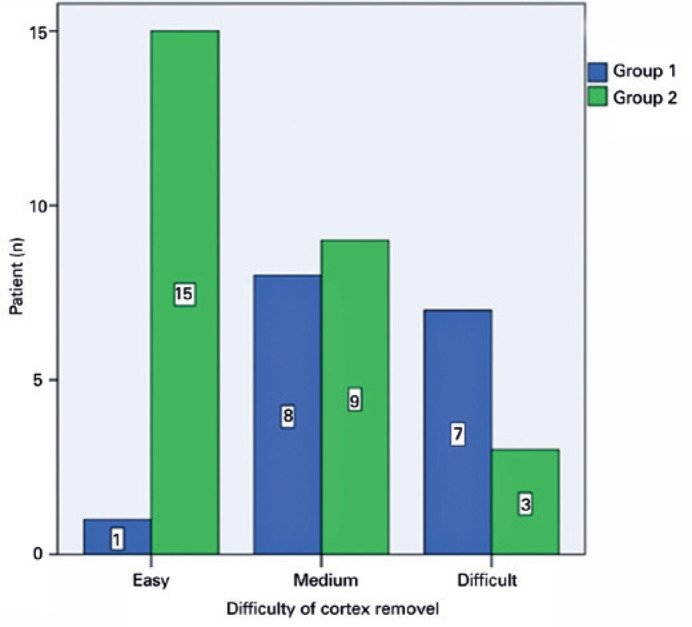
Difficulty of cortex removal.

Iris hooks were used as capsule retractors in five patients (31.3%) in group 1 and eight patients (29.6%) in group 2, with no statistically significant difference between the groups (p=0.91). There was also no significant difference between the groups in the difficulty of CTR implantation (p=0.124) (Figure 1). However, there was a significant difference between the groups in the difficulty of cortex removal (p=0.003) (Figure 2). Intrao perative complications were observed in 3 patients (18.7%) in group 1 and 11 patients (40.7%) in group 2, but there was no significant difference between the groups (p=0.18). In group 1, two patients had surge and one patient had a capsular rupture at the equatorial region. The patient with a capsular rupture underwent IOL implantation to the ciliary sulcus. In group 2, posterior capsule fluctuation was observed in eight patients (29.5%), and as a result, there was a posterior capsule rupture in two of these patients, a surge in two patients, and difficulty in capsulorhexis in one patient. Two patients with a posterior capsule rupture underwent IOL implantation without CTR implantation to the ciliary sulcus after an anterior vitrectomy.

## DİSCUSSİON

Subluxed lenses and zonular weakness can result from trauma, previous surgery, mature cataracts, high myopia, and PXF. PXF syn drome is the most common cause of zonular weakness^(11)^. Therefore, we included only patients with PXF syndrome in our study to ensure homogeneity and to obtain patients more quickly. The CTR has been of significant value in the management of cataract surgeries with zonular weakness. It can provide excellent capsule bag support to absorb tension from existing zonules and support areas of weakness^(3)^. After a capsulorhexis is performed, the CTR can be implanted at any step of phacoemulsification; however, the optimal timing remains controversial^(12)^. Unfortunately, only a limited number of studies have compared early and late CTR implantation.

Some authors advise early CTR implantation because it supports the area of zonular weakness and distributes the forces equally over all zonular areas^(4,13,14)^. When a CTR is placed prior to lens extraction, the capsular bag remains supported during the most critical intraoperative steps^(3)^. Ahmed et al. evaluated the optimal timing of CTR implantation in cadaver eyes^(3)^. They implanted the CTR early in two eyes and late in two eyes. They reported that early implantation of the CTR before lens extraction was associated with a marked increase in capsular bag displacement and zonular elongation compared with late implantation. Jacob et al. reported a 9.5% incidence of a clinically significant extension of zonular dialysis with the use of CTRs in 21 eyes with mild to moderate zonular dialysis in which the CTR was implanted before phacoemulsification^(13)^. Another problem with early CTR placement is that it can make cortical removal more challenging and tedious. In our study, there were no increases in capsular bag displacement and zonular weakness in either group.

Other authors advise performing late CTR implantation. Rai et al. implanted CTRs after lens extraction and had no cases of extension of dialysis during CTR implantation^(15)^. Ahmed et al. showed that late CTR implantation after lens extraction resulted in minimal capsular bag displacement and zonular stress in cadaver eyes^(3)^. The nucleus and cortex can also be easily removed without becoming trapped between the CTR and the capsular bag. In our study, cortex removal was significantly easier in patients who underwent late CTR implantation (p=0.003). A previous study reported that it was difficult to place a CTR before lens extraction in such dense cataracts, and further zonular dehiscence was found due to added stress on the intact zonules when the CTR was rotated into the capsular bag^(14)^. In the present study, there was no significant difference between the groups in the difficulty of CTR implantation (p=0.124).

Rai et al. reported late CTR implantations in 45 patients. In all cases, the IOL was placed in the capsular bag, which was well centered in most patients. Mild decentration of the IOL was detected in three patients (6.66%) at 6‑month follow‑up, without any subjective visual complaints^(15)^. Tribus et al. reported on 69 eyes with CTR implantation; however, the timing of the CTR implantation was not defined^(5)^. IOL implantation was performed in the capsular bag in 61 eyes (90%) and in the ciliary sulcus in 5 eyes (7%). In our study, most of the patients in both groups underwent IOL implantation in the capsular bag, while one patient (6.5%) in group 1 and two patients (7.5%) in group 2 underwent IOL implantation in the ciliary sulcus. At the end of the surgery, IOL centralization was observed in all patients. This may have resulted from the absence of any preoperative lens dislocation in our patients and zonular weakness in all quadrants rather than localized zonular dialysis.

Since the usage of the CTR, several complications associated with the CTR have been reported. These include accidental anterior capsule tears during CTR implantation, posterior dislocations of the CTR, and intraoperative dislocations after early CTR implantation in the capsular bag^(16^–^18)^. In the present study, there was no significant difference between the groups in the number of patients who had an intraoperative complication (p=0.18). In group 1, two patients had surge and one patient had a capsular rupture at the equatorial region. In group 2, posterior capsule fluctuations were observed in eight patients (29.5%), resulting in posterior capsule rupture in two of these patients, surge in two patients, and difficulty in capsulorhexis in one patient.

This study has some limitations. A better comparison could have been made with more participants. The degree of zonular weakness and the difficulty of CTR implantation and cortex removal were evaluated subjectively, which may have caused some bias. We used the effects of CTR during surgery and intraoperative complications in the evaluation of the optimal timing for the CTR implantation. Furthermore, more detailed information could have been obtained by comparing the long‑term postoperative effects.

In conclusion, cataract surgery in eyes with zonular weakness is technically challenging and time‑consuming. The rates of difficulty of implantation and of intraoperative complications were similar for early and late CTR implantation, whereas cortex removal was significantly easier with late CTR implantation. It should be kept in mind that in patients with PXF syndrome, cortex removal is more difficult with early CTR implantation and posterior capsule fluctuation may cause problems with late CTR implantation. The surgeon must consider the risk‑to‑benefit ratio of early versus late insertion for the optimal timing of CTR implantation.
